# Cholera prevention and control in Asian countries

**DOI:** 10.1186/s12919-018-0158-1

**Published:** 2018-12-07

**Authors:** Mesbah Uddin Ahmed, Mario Baquilod, Claudio Deola, Nguyen Dong Tu, Dang Duc Anh, Cindy Grasso, Anu Gautam, Wan Mansor Hamzah, Seng Heng, Sopon Iamsirithaworn, Musal Kadim, S. K. Kar, Mai Le Thi Quynh, Anna Lena Lopez, Julia Lynch, Iqbal Memon, Martin Mengel, Vu Ngoc Long, Basu Dev Pandey, Firdausi Quadri, Mitra Saadatian-Elahi, Sanjukta Sen Gupta, Ashraf Sultan, Dipika Sur, Dang Quang Tan, Hoang Thi Thu Ha, Nguyen Tran Hein, Phan Trong Lan, Shyam Raj Upreti, Hubert Endtz, N. K. Ganguly, Dominique Legros, Valentina Picot, G. Balakrish Nair

**Affiliations:** 1 Bangladesh Pediatric Association, Shahbag, Dhaka, Bangladesh; 2 Ministry of Health, Manila, Philippines; 3 Save the Children, London, UK; 4 National Institute of Hygiene and Epidemiology, Hanoi, Vietnam; 5 Fondation Mérieux, 17 rue Bourgelat, 69002 Lyon, France; 6 UNICEF, Bangkok, Thailand; 7 Ministry of Health, Kuala Lumpur, Malaysia; 8 Ministry of Health Cambodia, Phnom Penh, Cambodia; 9 Ministry of Health, Bangkok, Thailand; 10 Indonesia Pediatric Society, Jakarta, Indonesia; 11 S’O’A University, Bhubaneswar, Odisha India; 12 Delivering Oral Vaccine Effectively, Manila, Philippines; 13 International Vaccine Institute, Seoul, South Korea; 14 Pakistan Pediatric Association, Karachi, Pakistan; 15 Agence de Médecine Préventive, Madrid, Spain; 16 Ministry of Health, Hanoi, Vietnam; 17 Ministry of Health, Kathmandu, Nepal; 18 International Centre for Diarrhoeal Disease Research (icddr,b), Dhaka, Bangladesh; 19 Hopital Edouard Herriot, Hospices Civils de Lyon, Lyon, France; 20 Translational Health Science and Technology Institute, Pali, Haryana India; 21 Mid city Hospital, Lahore, Pakistan; 22 Program for Appropriate Technology in Health (PATH), New Delhi, India; 23 Institut Pasteur, Hô-Chi-Minh, Vietnam; 24 Group for Technical Assistance, Kathmandu, Nepal; 25 World Health Organization, Geneva, Switzerland; 26 World Health Organization, New Delhi, India

**Keywords:** Cholera, Asia, Water, Sanitation and hygiene (WASH), Cholera vaccine, IDEA

## Abstract

Cholera remains a major public health problem in many countries. Poor sanitation and inappropriate clean water supply, insufficient health literacy and community mobilization, absence of national plans and cross-border collaborations are major factors impeding optimal control of cholera in endemic countries.

In March 2017, a group of experts from 10 Asian cholera-prone countries that belong to the Initiative against Diarrheal and Enteric Diseases in Africa and Asia (IDEA), together with representatives from the World Health Organization, the US National Institutes of Health, International Vaccine Institute, Agence de médecine préventive, NGOs (Save the Children) and UNICEF, met in Hanoi (Vietnam) to share progress in terms of prevention and control interventions on water, sanitation and hygiene (WASH), surveillance and oral cholera vaccine use.

This paper reports on the country situation, gaps identified in terms of cholera prevention and control and strategic interventions to bridge these gaps.

## Background

Cholera represents an important public health problem in many settings. Annually, 2.8 million cases and 91,500 deaths occur in cholera endemic countries [1]. Beyond direct health concerns, cholera also presents a significant economic burden [2].

In addition to poor sanitation and inappropriate clean water supply, insufficient health literacy and community mobilization, absence of national plans and cross-border collaborations are major factors impeding optimal control of cholera in endemic countries. Poor knowledge of the real burden of cholera due to substantial under-reporting is also another obstacle [3–5]. Potential factors which will worsen the situation in the coming years are climate change, urbanization, increase in population density and, (further) rise of social inequalities [6].

Progress towards better hygiene and sanitation will be faster if a multidisciplinary and multi-sectoral approach is developed and implemented. Implementation of such strategy requires action under two key pillars: 1) increase political and financial support for cholera control and; 2) strengthen multi-sectoral cholera prevention and control programs.

In accordance with these pillars, the Initiative against Diarrheal and Enteric Diseases in Africa and Asia (IDEA) was born in 2011. IDEA is an independent and multidisciplinary network of professionals from cholera-prone countries in Asia and Africa, in collaboration with national and international stakeholders. IDEA’s main goal is to facilitate and support the implementation of relevant prevention and control interventions on water, sanitation and hygiene (WASH), and on the use of oral cholera vaccine (OCV) by sharing information and best practices and to raise awareness on the country specific cholera situation. Between 2015 and 2016, four IDEA workshops have been successfully achieved in Asia and Africa. The fifth IDEA meeting took place in Vietnam (Hanoi, 6–9 March 2017) and involved experts from 10 Asian cholera-prone countries (Bangladesh, Cambodia, India, Indonesia, Malaysia, Nepal, Philippines, Pakistan, Thailand, and Vietnam) together with representatives from the WHO, the US National Institutes of Health, International Vaccine Institute, Agence de médecine préventive, NGOs (Save the Children, StC), and UNICEF.

Country representatives shared their respective country situation, and progress in terms of WASH, surveillance and OCV use. Representatives from different health agencies provided an overview of available initiatives, interventions and tools in Asia. Following the plenary sessions, participants worked in subgroups to identify gaps in terms of cholera prevention and control and to discuss strategic interventions to bridge these gaps.

## Country situation

Update on cholera epidemiology, progress in the prevention and control of cholera and a mapping of country capacities were presented (Table 1). Suboptimal WASH including lack of safe water supply, appropriate sanitation facilities and persistence of open defecation were among factors that contribute to persistent cholera outbreaks. OCV has been used in Bangladesh, India, and Nepal but is not included in the National Immunization Programs. Cholera surveillance systems are in place in all participating countries but the type of surveillance and the extent of coverage differ considerably between countries. Awareness campaigns and community mobilization are regularly conducted in order to sensitize the public to simple preventive measures. Each country faces several challenges but improving WASH and increasing the coverage areas of surveillance systems were commonly reported.

**Table 1:** Summary of country situation update as reported by country representatives

Thematic/Country	Bangladesh	Cambodia	India	Indonesia
Epidemiology	Endemic, and seasonal outbreaks CFR 25 to 50% without treatment and 1% with treatment	Not cholera endemic, sporadic cases in 18 provinces. CFR <1% but higher in hard to reach villages	Endemic with an estimated 834,00 cholera cases and 25,000 deaths every year Several states do not report any cholera cases potentially due to limited surveillance system	Low endemic. No outbreaks since 2011 Incidence of diarrheal diseases for all age population is 350/1000 population and 670/ 1000 children < 5 years old
WASH	Lack of water pipe	Data not available	Open defecation with limited availability of safe drinking water supply mainly in rural area Sharing sanitation facilities with other households	Practice of healthy and hygienic behavior by only 38.7% Open defecation practiced by 9.4% population (2.5 millions), 10.9% use unsafe water, 7.3% drinks uncooked water
OCV vaccination	OCV included in national plan for at risk groups. Technology transfer for vaccine development in country Locally produced Vaccine (Cholvax) will be implemented	None	Vaccine introduction study done in Odisha state OCV vaccine is not part of the EPI program	None
Surveillance/Diagnostic	Hospital based surveillance at 2% Nationwide surveillance on- going at 21 sites	Event based surveillance, laboratory confirmed (CamEWARN) Surveillance of Acute Watery Diarrhea through CAM EWARN Reporting of laboratory confirmed cholera cases to CDC Dept Outbreak investigations	Weekly surveillance system in all regions Guidelines and SOP for early case detection Global positioning system & Google Earth in the investigation cholera outbreak. Continuous laboratory surveillance of ADD in all districts Visit of collector to affected sites for monitoring quick action	
Advocacy	Advocacy meeting: January 2017	Communications (TV and radio spots, posters, flyers) Community mobilization	Sensitization of PRI members, local PHC staff, ASHA, AWW and community Other modes of community mobilization such as interpersonal Communication by door-to-door visit	
Challenges	Licensure and funding for locally produced vaccine (Cholvax) deployment, with WHO pre-qualification Inadequate coverage of the surveillance system	Under-reporting of surveillance systems	Marginalized rural and tribal populations Poor availability of safe drinking water supply Inadequate ownership of programs Poor local health infrastructure Inadequate priority setting mechanisms Long incubation periods for research programs Introduction of OCV	Under-reporting Lack of RDT
Thematic/Country	Malaysia	Nepal		Pakistan
Epidemiology	Not Endemic except in Sabah region Incidence rate <1 per 100,000 populations and CFR <1% in recent years Malaysian: Foreigners incidence rate = 80:20. Cycle pick every 3 years	Endemic, frequent outbreaks mainly during rainy season 5042 ADD cases and 169 laboratory confirmed Cholera cases reported mainly from Kathmandu valley (150/169). No deaths occurred.		Endemic 4-6 episodes of diarrhea per child per year < 5 year Under five deaths per year from Diarrhea: 13.2%
WASH	Scarce safe water supply in some areas Unresolved environmental issues – excreta, solid waste Poor hygiene & food sanitation with cross border crossing and illegal coastal and urban settlements	Suboptimal WASH status Basic Water Supply coverage: 83.59%, Sanitation: 87.17%. Hand-washing: 72.5%		16M do not have access to clean drinking water 27% consume tap water, 86% have access to improved water source, 73% have access to sanitation facilities 13% no toilet facility
OCV vaccination	Vaccine and antimicrobial prophylaxis OCV not in EPI but available in the private health facilities Oral prophylaxis for close contacts and food handlers	Reactive OCV Vaccination in Rautahat district in 2014. Preventive OCV vaccination campaign in Nuwakot and Dhading in 2015 and in Banke district in 2016		OCV not registered
Surveillance/diagnostic	Mandatory web based within 24-hour notification National guidelines and laboratory diagnostic capacity in all laboratories Regulatory Infrastructure	Cholera is an EWARS reportable disease. Clinical cases reported monthly from health facilities through existing HMIS system. Cholera Surveillance embedded in the existing AMR sentinel surveillance system using 18 sites. Comprehensive Targeted Interventions (CTI) to Control Cholera in Kathmandu Valley in Kathmandu valley in 2016		Facility based surveillance system in place in the province of Punjab since 2011(which has 60% of the population of Pakistan limited laboratory capacity Passive case- based surveillance from large hospitals of major cities, and WHO EMRO Documents and reports of NGOs working in disaster situations
Advocacy	Political commitment, interagency collaboration and coordination Legal approach for child education, case notification and management, food sanitation Subsidy for the poor (rural and urban) Ensure accessibility to affordable healthcare and education Free treatment and quarantine leave for working parents Restructure settlements with affordable homes Hygiene Promotion, community engagement, Social Mobilization campaign adapted to local culture	Door to Door Awareness Campaign. Community Level Intervention: Booth Campaigns – Strategic Locations -Awareness rallies -Miking (In mobile vehicle and also during rallies) -Awareness sessions to community groups and key community actors -Food and food outlet inspection- Food authority and Municipality -Mass communication by various media and special programs -Schools reached to educate and use children on Cholera and prevention		
Challenges	Cross border crossing Illegal coastal and urban poor settlements Poverty, illiteracy and language barrier Inadequate financial investment for WASH	Identify risk groups and target mass vaccination by strengthening surveillance. Need to give high priority to improve WASH status. Enhance collaboration and coordination. Advocacy needed to introduce the OCV vaccination Endorse Cholera Prevention and Response National Road Map		Recurrent humanitarian emergencies Weak surveillance system and underreporting Limited laboratory capacity Under resources of the public health control activities Poor water and sanitation condition in conflict affected countries Lack of cross border collaboration between the neighboring countries
Thematic/Country	Philippines	Thailand		Vietnam
Epidemiology	14,592 diarrheal and 96 deaths cases in 2016 124 (0.85%) were laboratory confirmed cholera No deaths.	Incidence significantly decreased in the past decades while outbreaks occasionally occurred: 4 outbreaks since 2017 125 cases in 2015 Main transmitters: Employees of the seafood industry, Migrant population No cholera outbreaks post-flood disasters in recent years		No Cholera since 2012
WASH	Zero Open Defecation Program. Environmental Health Program: WASH, Regional Sanitary Engineers, Local Sanitary Inspectors	100% toilets at all houses Sewage management Chlorinated tap water and/or bottle water		Health education Clean water supplies and Environment sanitation Food hygiene and safety
OCV vaccination		OCV in a special setting, population in the temporary shelter at Thai-Myanmar Border		Local vaccine production NRA approved by QWHO; Vaccination deployed in 16 provinces with high incidence and for high risk areas and populations
Surveillance/diagnostic	Event-based Surveillance Epidemiology Bureau of the DOH, Program Manager Regional Epidemiology & Surveillance Units Regional Program Coordinators Collection of human (rectal swab, stool) and environmental (water) samples. Laboratory testing of water samples thru the use of Colilert machine. Records review and active case finding Random inspection of water refilling stations Continuous surveillance of diarrhea cases Food & Water-borne Program. Regional Sanitary Engineers Local Sanitary Inspectors	Hospital-based surveillance system – Early detection of suspected cholera cases – Laboratory confirmation • Timely and proper management of patients • Prompt investigation and control by the trained Surveillance and rapid response teams (SRRTs). Improving Sanitation and Chlorination of Water Supply 12 Regional Laboratory Centers of department of Medical Sciences. Water and Food samples with 1% APW		National guidelines for cholera diagnosis, treatment, surveillance, response, control and prevention Testing in dog slaughter houses and restaurants Mobile teams for early detection and investigation of outbreaks Urgent reporting to higher level of health care system Close collaboration between treatment and preventive systems in reporting, specimen collection, and sharing specimen Laboratory testing at national and regional Level At district level: Specimen collection, storage and transportation; Microscope examination, Gram staining, Testing of water, fresh vegetables in restaurants and markets
Advocacy	The Department of Health (DOH) recognized the distinctive link between sanitation and better health, need for a new vision in sanitation, expressed in clearer policy and action programs			Enhance the leadership of political system and of Local Steering Committee on cholera prevention and control Mobilize whole political system in cholera prevention and control. Close collaboration between related sectors on food hygiene and safety, clean water supply and environmental sanitation, education, information, transportation
Challenges		Continuing improvement of sanitation and safe water Limited vaccination. Varying capacity in diagnosis and treatment.		Maintaining and improving the clean water supply and environment sanitation program. Strengthen collaboration among neighboring countries on sharing information and cholera control

## Existing interventions on cholera prevention and control in Asia

UNICEF chairs the WASH working group of the Global Task Force on Cholera Control (GTFCC). The WASH-GTFCC working group has developed technical briefs and set-up a study to estimate the effectiveness of households’ disinfection practices.

WASH is also one of the main actions of StC, an international non-governmental organization that promotes children’s rights. The StC global approach to cholera includes emergency health units, prepositioning stocks in eight countries, and a multi-sectoral approach. The objectives are to i) keep fecal matter away from drinking water, ii) inactivate cholera in contaminated water and iii) provide WASH facilities for medical teams and patients.

Another significant preventive tool available now is the global stockpile of OCV that was created in 2013 as an additional tool to help control cholera epidemics [7]. The WHO, UNICEF, and the Delivering Oral Vaccine Effectively (DOVE) project work in close collaboration to ensure that at-risk populations will benefit from OCV in an appropriate and effective manner.

The dynamic creation by the establishment of stockpile has played a key role in increased use of OCV [8–11] (Table 2). However, vaccine availability remains a major barrier limiting mass vaccination interventions. Two campaigns were conducted in South Sudan (2015) and in Zambia (2016) to evaluate the efficacy of a single dose strategy during outbreaks. The results showed that vaccinating twice the number of people with a single dose can prevent more cases and deaths during an outbreak by providing rapid herd protection. Similar findings have been provided by a modeling study that assessed the impact of one-dose OCV versus 2-doses in outbreak settings [12].

**Table 2:** The use of oral cholera vaccine stockpile in 2013–2016

Year	Type of Campaign	Number	Country
2013	Endemic	2	Haiti (2)
2014	Endemic	10	DRC, Guinea, Haiti (8)
	Humanitarian crisis	7	South Sudan, Ethiopia
2015	Outbreak	4	Malawi, South Soudan, Iraq, Nepal
	Humanitarian crisis	6	South Sudan (3), Tanzania, Cameroon, Malawi
2016	Endemic	1	Haiti
	Humanitarian crisis	3	Niger, South Sudan (2)
	Outbreak	2	Malawi, Zambia

Other novel strategies including self-administration of the second dose (fisherman living in floating homes), out of cold chain use during distribution (Guinea 2012) and OCV delivery combined with other interventions (Refugee camps, Cameroon 2015) have also been tested and provide evidence of the feasibility of conducting OCV campaigns in a variety of scenarios.

To help developing country vaccine manufacturers, the International Vaccine Institute (IVI) engaged in a technology transfer development strategy (Table 3). Long-term efficacy of Shanchol [13] and safety and immunogenicity of Euvichol [14] have already been assessed. Cholvax is currently under evaluation in a non-inferiority trial to Shanchol in Bangladesh. In parallel, an individually randomized placebo-controlled trial to evaluate the use of a single dose in an endemic setting was completed [15].

**Table 3:** List of oral cholera vaccine technology transfer by the International Vaccine Institute

Company (Country)	Vaccine	Partnership	Stage of development
Vabiotech (Vietnam)	mORCVAX	IVI re-formulated redeveloped the process to meet WHO standards	Licensed in Vietnam
Shanat (India)	Shancol	Technology transfer May 2008	Licensed in India (Feb 2009)
			WHO prequalified Sep 2011
Eubiologics (Korea)	Euvichol	Technology transfer May 2010–2011	Korean export license 2014
			WHO prequalified Dec 2015
Incepta (Bangladesh)	Cholvax	Technology transfer May 2014	IVI conducting clinical trial in Bangladesh, license expected in 2017–2018

## Workshop session

**Fig. 1:**
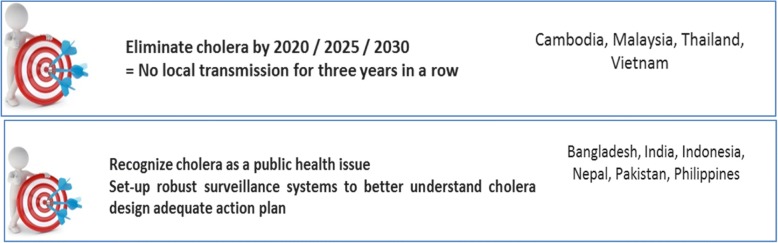
Countries’ aims for cholera prevention and control

To elicit more consideration for the prevention and control of cholera in participating countries, a brainstorming breakout session was held. The first part of the session was focused on what should countries aim at in terms of cholera prevention and control. There were two clusters of countries in terms of mid-term objectives depending on where they currently stand in cholera prevention and control (Fig. 1). Cambodia, Malaysia, Thailand and Vietnam aim at eliminating cholera in the coming years while recognizing cholera as a public health problem was the main mid-term objective for others.

Participants identified five main areas of strategic intervention to bridge the gaps and hence to reach the objectives of countries in terms of cholera prevention and control.

### Implementation/reinforcement of surveillance systems (Fig. 2)

Currently, surveillance systems are patchy or minimal. Countries must strengthen the existing surveillance systems both in terms of coverage and capacity (e.g. laboratory diagnostic tests). This would allow early case detection and immediate response. Regular analysis and dissemination of data at the national and neighborhood level is also believed to act as a driver in the prevention and control of cholera.

**Fig. 2:**
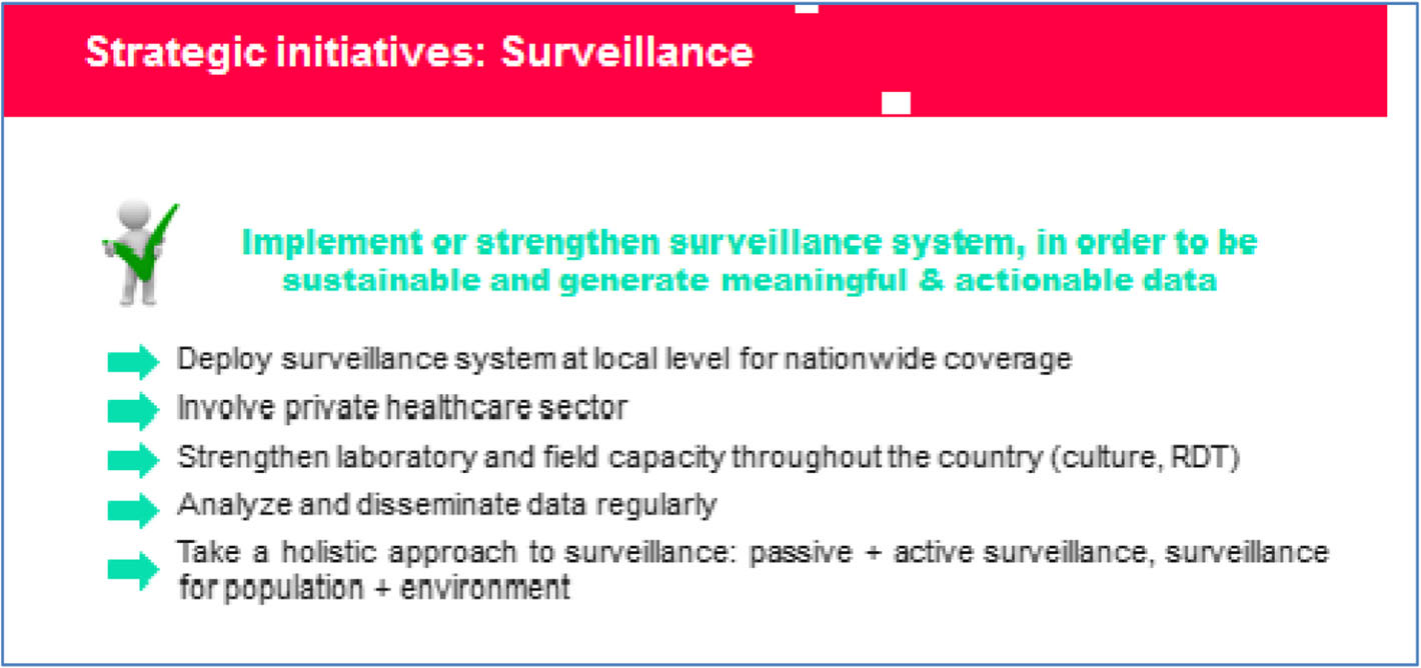
Implementation/reinforcement of surveillance systems

### Water, sanitation and hygiene promotion (Fig. 3)

WASH is universally recognized as a major component of preventing several infectious diseases [16]. Implementation of successful proactive WASH campaigns requires political will and community engagement. Tailored messages should be developed to increase awareness of open defection, food and environmental safety and hygienic practices. Special attention should be given to schools. Engagement of political leaders could help in funding WASH priorities and in implementing food and water safety laws.

**Fig. 3:**
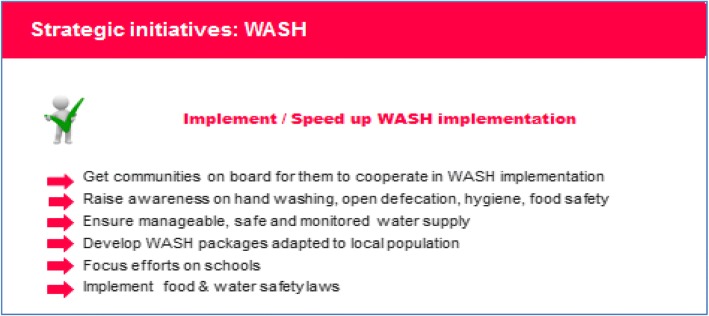
Water, Sanitation and Hygiene promotion

### Deployment of oral cholera vaccine (Fig. 4)

OCV is considered as a supplementary tool for cholera prevention and control [17]. Pre-emptive and reactive OCV vaccination programs in cholera hot spots in several African and Asian countries have shown promising results [9–11] and should be sustained. Cost-effectiveness analysis of mass cholera vaccination campaigns is a key consideration for optimizing OCV deployment.

**Fig. 4:**
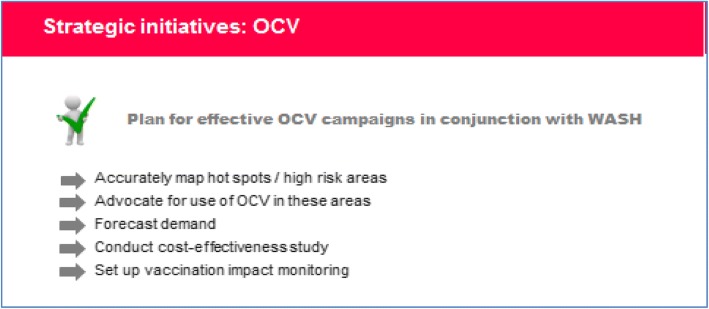
Deployment of oral cholera vaccine

### Social mobilization and health promotion (Fig. 5)

To be effective, community mobilization should be based on outreach and awareness campaigns that improve knowledge on the disease, prevention and existing treatment. They should provide transparent sharing of information and proper education about routes of transmission and prevention measures. Appropriate involvement of media and schools could ensure fast spread of the information.

**Fig. 5:**
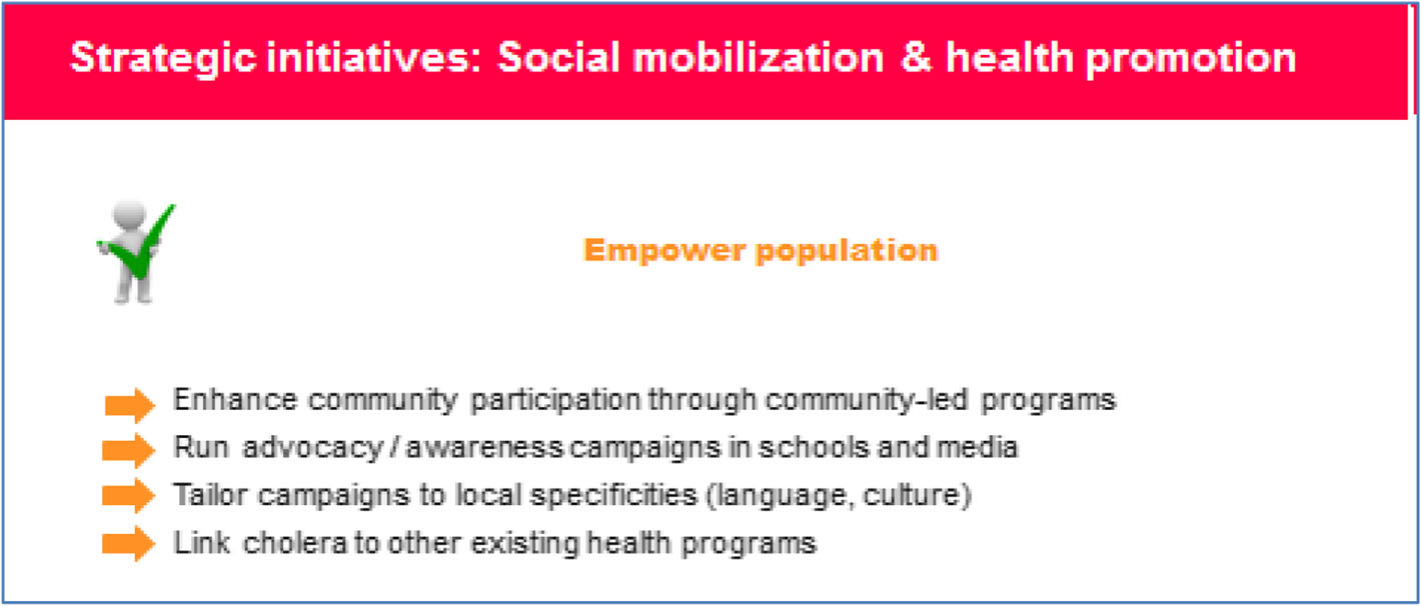
Social mobilization and health promotion

### Collaboration (Fig. 6)

Cholera preparedness and responses should include inter-sectoral partnership between health authorities at national and international level, civil society and other stakeholders. Cholera epidemics commonly occur in a cross-border manner, emphasizing the importance of cross-border cooperation to control and prevent the spread of the disease.

**Fig. 6:**
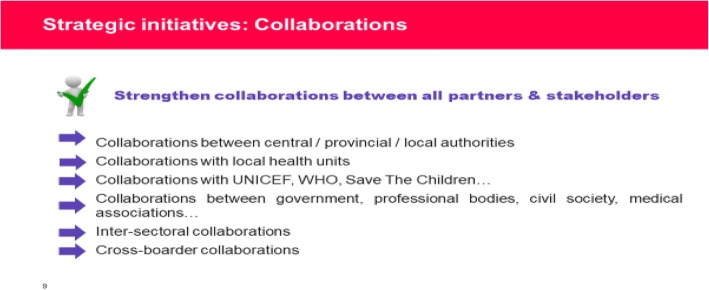
Collaboration

## Conclusions

Cholera remains a continuous threat with high health and economic burden in several South Asian countries. Despite tremendous efforts, prevention and control of cholera suffers from a number of challenges and issues in Asia. Inadequate WASH was identified as a major barrier in the prevention and control of cholera. Countries believe that WASH responses were often reactive and the criteria to trigger WASH responses were often unclear. Funding of WASH priorities remains also a challenge. This might be due to the difficulties related to measuring quantitatively the effectiveness and sustainability of WASH, as compared to vaccination which is precisely measured and evaluated using immunological or surveillance data, or directly by determining vaccination status. The group recommended that priority WASH interventions in emergency situation should include: i) increased water supply, ii) improved quality of water supplied, iii) increased access to excreta disposal facilities, solid waste collection and disposal, hand washing facilities, soap and water storage vessels and iv) hygiene education and social mobilization.

Weak surveillance systems, underreporting and limited laboratory capacities have been reported by country representatives who advocated for reinforcement of active and passive cholera surveillance system: capacity building, training, guidelines, and equipment facilities. Participating countries recommended that the Cholera Prevention and Response National Road Map should also be endorsed urgently.

OCV have the added advantage of herd protection which further decrease significantly the number of cases. Thanks to technology transfer, the OCV stockpile will grow with more vaccines being manufactured by different companies. Vaccine price could also be positively impacted by multiplying manufacturers. The group concluded that OCV should be introduced and used in different ways according to the country situation (special populations, integrated in the existing immunization programs or used in emergency situations). The use of one-dose OCV regimen could also be a promising solution during emergency situations. Other innovative OCV delivery strategies are also being tested. This includes:

- ✓ A self-administered second dose for the fishermen in “floating homes” living on Lake Chilwa is carried out by MSF. The second dose is given together with first dose that will be home-based self-administration,
- ✓ A community-led self-administrated second dose on the six islands of Lake Chilwa carried out by AMP. The second dose is given to community leaders and kept in large cool boxes to be administrated under direct observation of the leader.

Evidence supports that killed whole cell vaccines are stable at high temperature for long periods [9, 18, 19]. Therefore, vaccine can be kept under cold chain in central stock but used out of the cold chain during distribution in hard-to-reach areas.

Provision of necessary supply will have the greatest impact on cholera burden if it is coupled with educational programs, community engagement and mobilization. The efficacy of a number of actions (e.g. door-to-door visits, placards, slogans, banners, special annual campaign) has already been tested and ought to be sustained. Outbreaks should be investigated and controlled as rapidly as possible by means of communication. Low-cost nudges behavior changes with a preventive approach can could help to increase compliance to hand-washing. In a nudge-based intervention study (i.e. positive reinforcement to influence people’s behavior) carried in rural Bangladesh, hand-washing with soap increased from 4% at baseline to 68% the day after nudges were completed and 74% at 2 and 6 weeks post intervention [20].

Cholera still causes stigma as it is said to be a ‘forgotten disease’ mainly affecting ‘poor people’. Outreach meetings including public and private stakeholders and the general population are warranted to recognize that cholera is not only a health problem but also the direct consequence of poor WASH, linked to various environmental, climatic and socio-economic situations. Cholera can be prevented and controlled via complementary, synergistic and multidisciplinary interventions including access to safe water supply, end of open defection, increased hygiene, political engagement, community mobilization, prompt case management and vaccination.

## Perspective

Integrated multi-sectoral approaches have proven to be the best mechanism to implement effective strategies for the prevention and control of infectious diseases. Coordinated stakeholder activities are key components of disease control success. In this perspective Fondation Mérieux hosting organization along with present stakeholder during the meeting announces its full commitment to the coordinated strategy and join its cholera activities along with other partners within the Global Task Force on Cholera Control to implement the renewed strategy for cholera control while building on existing achievements.

## Abbreviations

GTFCC: Global Task Force on Cholera Control
IDEA: Initiative against Diarrheal and Enteric Diseases in Africa and Asia
OCV: Oral cholera vaccine
StC: Save the Children
WASH: Water, sanitation and hygiene
